# When the Introducer Becomes the Limiting Factor: A Technical Note on a Simple Modification to Facilitate Spinal Anesthesia With Pencil-Point Needles in Morbidly Obese Patients

**DOI:** 10.7759/cureus.114321

**Published:** 2026-08-11

**Authors:** Ergun Gunduz, Ayse Nur Yeksan, Seza Apiliogullari

**Affiliations:** 1 Anesthesiology and Critical Care, Istanbul Atlas University School of Medicine, Istanbul, TUR

**Keywords:** anesthesia spinal, difficult spinal anesthesia, morbid obesity, obstetric anesthesia, whitacre spinal needle

## Abstract

Morbid obesity is associated with increased skin-to-dura distance, which may complicate spinal anesthesia using standard-length pencil-point spinal needles. We describe a simple technical modification developed during spinal anesthesia in a parturient with a body mass index of 40 kg/m^2^, in whom a 27-gauge, 90-mm Whitacre-type spinal needle with the manufacturer-provided introducer was considered insufficient to reach the subarachnoid space. Comparison of the standard introducer with the hub of a conventional 10-mL syringe demonstrated a reduction of approximately 9 mm in external projection beyond the skin surface. The syringe hub was subsequently used as an alternative introducer, effectively increasing the usable length of the spinal needle without altering the needle itself or requiring additional equipment. Awareness of introducer-related reductions in effective working length may help clinicians avoid unnecessary procedure failure or escalation to more invasive alternatives when longer spinal needles are unavailable or impractical.

## Introduction

Pencil-point spinal needles have an atraumatic, non-cutting tip and are typically used with an introducer needle [1,2]. Compared with cutting-point needles, they cause less tissue trauma and are associated with a lower incidence of post-dural puncture complications, making them the preferred choice in many clinical settings [1,2]. In morbidly obese patients, increased skin-to-dura distance may limit successful spinal anesthesia with standard-length pencil-point spinal needles [3,4]. While attention is usually directed toward selecting longer spinal needles in these situations, the potential contribution of the introducer to effective needle length is rarely considered [3].

Pencil-point spinal needles require an introducer because their blunt, non-cutting tip and relatively flexible shaft make direct advancement through the skin and subcutaneous tissues difficult [2]. The introducer provides stability and facilitates accurate needle guidance [2]; however, its hub reduces the effective working length of the spinal needle [3]. Although this reduction amounts to only a few millimeters, it may become clinically relevant in patients with increased tissue depth. Corfe [3] previously reported that differences in introducer hub design between commercially available spinal needle systems reduced the effective working length of the spinal needle sufficiently to prevent dural puncture in obese obstetric patients. To overcome this limitation, the author changed to a spinal needle system with a shorter introducer hub [3].

Accordingly, the aim of this technical note is to describe a practical modification of the standard introducer assembly designed to increase the effective working length of pencil-point spinal needles and potentially facilitate spinal anesthesia in situations where additional needle reach may be beneficial.

## Technical report

A patient with a body mass index (BMI) of 40 kg/m^2^ was scheduled to undergo surgery under spinal anesthesia using a 27-gauge, 90-mm pencil-point spinal needle together with the manufacturer-provided introducer (Egemen International, Izmir, Turkey). During the procedure, the available needle length was considered insufficient for further advancement toward the subarachnoid space.

Inspection of the spinal needle-introducer assembly suggested that the hub of the introducer occupied a meaningful proportion of the available needle length outside the patient and therefore reduced the effective working length of the spinal needle.

**Figure 1:**
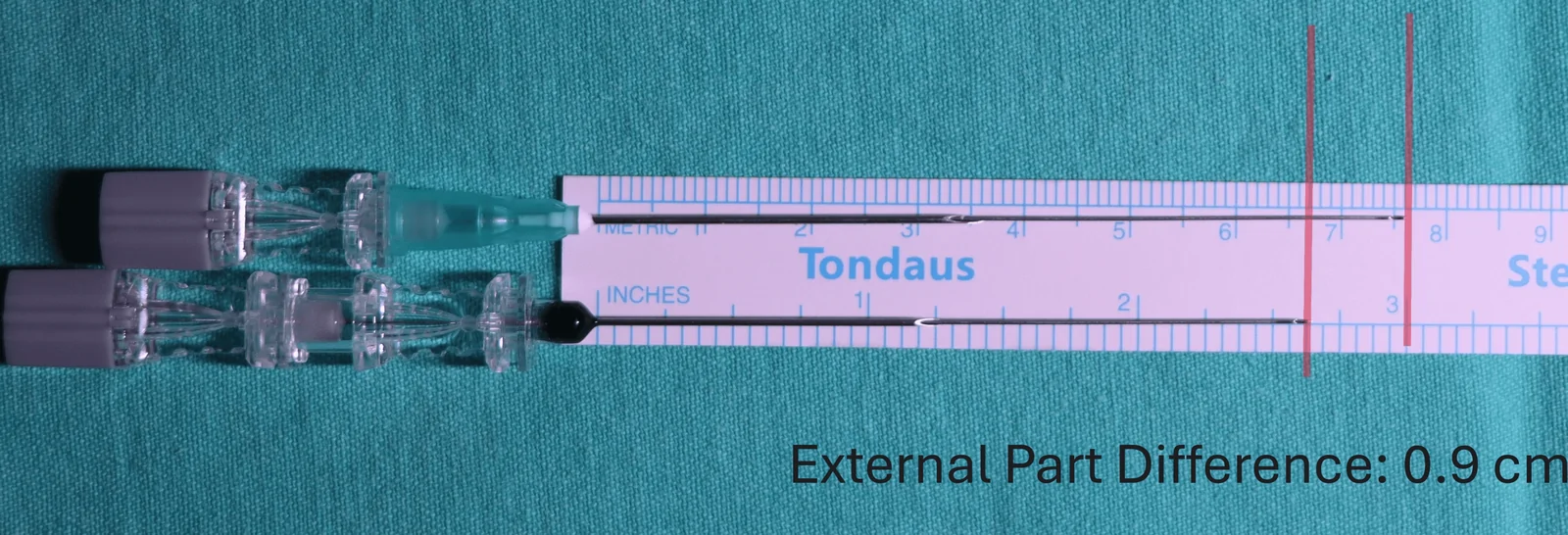
Comparison of the standard introducer and the syringe-hub introducer configurations demonstrating the reduction in external part (hub) length and the resulting increase in effective spinal needle working length.

**Figure 2:**
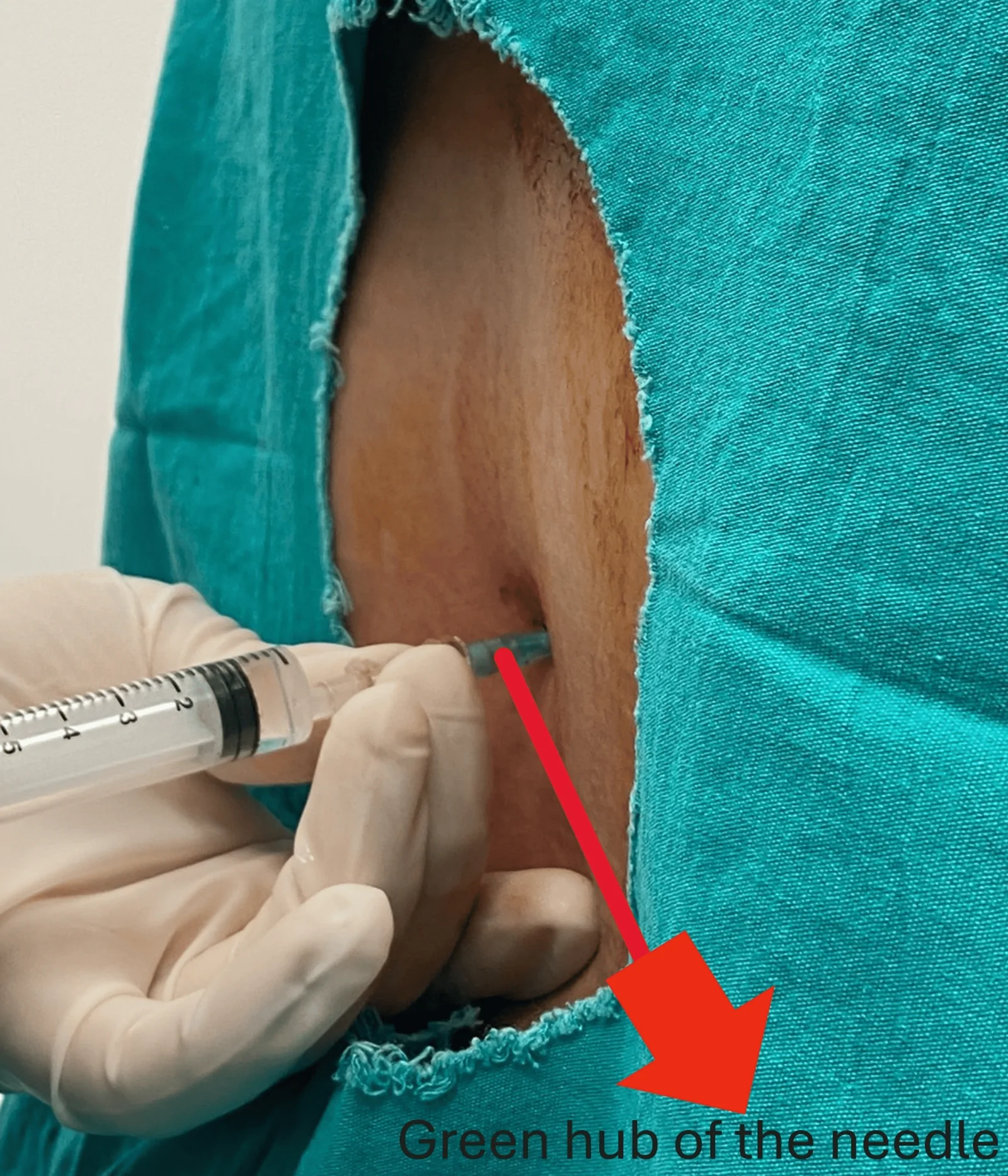
Clinical application of the modified introducer technique during spinal anesthesia. The image demonstrates the syringe-hub introducer configuration with the pencil-point spinal needle during the spinal block procedure.

To overcome this limitation, we considered whether reducing the hub length could increase the available working length of the spinal needle. Measurement of the exposed needle lengths demonstrated that the syringe-hub configuration provided an additional 0.9 cm of exposed spinal needle length compared with the standard introducer configuration (Figure 1). In routine clinical practice, an additional 0.9 cm of effective needle length may be decisive in achieving successful dural puncture, especially in patients with obesity, marked subcutaneous tissue thickness, or an increased skin-to-subarachnoid distance. The syringe hub was therefore used as an alternative introducer, effectively increasing the usable length of the spinal needle without altering the spinal needle itself or requiring specialized equipment. The clinical application of the modified introducer technique is shown in Figure 2.

## Discussion

Alternative approaches for anticipated difficult spinal access in obese patients include the use of longer spinal needles [3], ultrasound-guided neuraxial techniques [5], and paramedian approaches [6]. In our practice, another possible option was the use of the 138.5-mm pencil-point spinal needle included in a combined spinal-epidural (CSE) set (B. Braun Melsungen AG, Melsungen, Germany; Table 1).

**Table 1:** Comparison of available approaches for neuraxial anesthesia in patients with increased tissue depth.

Technique	Advantages	Limitations
Modified introducer technique (this report)	Simple, low-cost modification that may increase effective needle length when longer spinal needles are unavailable	Limited clinical experience; potential concerns regarding stability, sterility, and off-label use
Longer spinal needles	Specifically designed for patients with increased skin-to-dura distance	May not be available in all centers; may be more difficult to control
Ultrasound-guided neuraxial anesthesia	Helps identify landmarks and estimate depth before needle insertion	Requires equipment and operator experience
Paramedian approach	Provides an alternative route when midline access is difficult	Requires experience and may still be challenging in severe obesity
Combined spinal-epidural (CSE) technique	Allows spinal anesthesia with the option of extending the block	More complex and requires additional equipment

In our case, a 120-mm spinal needle was not available at the time of the procedure. Ultrasound guidance may help identify the shortest skin-to-dura trajectory; however, it requires operator expertise and was not routinely utilized in our practice at the time of the procedure. Similarly, although paramedian approaches may facilitate neuraxial access in patients with difficult anatomy or poorly defined landmarks [6], they may not overcome situations in which the effective working length of the spinal needle itself becomes the limiting factor. Therefore, we preferred the modified introducer technique, which had previously been used successfully in our practice as a rescue maneuver in similar situations.

Our observation highlights an additional and potentially modifiable factor that is rarely considered during difficult spinal anesthesia: the introducer hub itself may become the limiting factor rather than the spinal needle length.

This technical note has several limitations. First, the described modification was applied in a single clinical case; therefore, conclusions regarding its generalizability, safety, and clinical effectiveness cannot be established. Second, unlike manufacturer-provided introducers, a syringe hub is not specifically designed or validated for neuraxial needle guidance. Previous studies have demonstrated that introducer design, gauge, and bevel orientation may influence spinal needle deflection and directional control [7]. Therefore, although the modification increased the available working length in our case, potential effects on needle stability, alignment, and advancement characteristics should be considered. Further studies are required to evaluate the reproducibility, safety, and clinical applicability of this approach.

## Conclusions

The proposed modification does not represent the development of a new spinal needle system but rather a simple adjustment of the existing introducer configuration using readily available materials. By increasing the effective working length of standard pencil-point spinal needles, this technique may provide a practical rescue option in selected cases where longer spinal needles are unavailable.
